# Oxymel: A systematic review of preclinical and clinical studies

**DOI:** 10.1016/j.heliyon.2023.e22649

**Published:** 2023-11-21

**Authors:** Narges Sharifi Darani, Mahdi Alizadeh Vaghasloo, Asma Kazemi, Hakima Amri, Thomas Rampp, Mohammad Hashem Hashempur

**Affiliations:** a Persian Medicine Network (PMN), Universal Scientific Education and Research Network (USERN), Tehran, Iran; b Department of Traditional Medicine, School of Persian Medicine, Tehran University of Medical Sciences, Tehran, Iran; c Nutrition Research Center, School of Nutrition and Food Sciences, Shiraz University of Medical Sciences, Shiraz, Iran; d Department of Biochemistry and Cellular & Molecular Biology, Division of Integrative Physiology, Division of Whole Person Health and Wellness, Georgetown University Medical Center, Washington, DC, USA; e Department of Integrative Medicine, University of Duisburg-Essen, Duisburg, Germany; f Research Center for Traditional Medicine and History of Medicine, Department of Persian Medicine, School of Medicine, Shiraz University of Medical Sciences, Shiraz, Iran

**Keywords:** Oxymel, *Serkangabin*, *Sekanjabin*, Functional food, Traditional Persian medicine, Complementary medicine

## Abstract

**Background:**

Oxymel is a functional beverage with a rich historical background of use in multiple societies. Various simple and compound oxymels are prescribed in certain complementary and traditional medical systems, including traditional Persian Medicine. In recent years, numerous clinical and preclinical studies have been conducted in the pharmacy and food industry to investigate the efficacy of various oxymel formulations. This article aims to systematically review and summarize interventional studies on oxymel in both clinical research and animal models.

**Methods:**

Relevant articles were searched in Embase, MEDLINE, Web of Science Core Collection, Scopus, and Google Scholar from inception to July 2023 using the keyword “Oxymel” and its equivalents in other languages. Animal and human interventional studies were selected from the search results for review.

**Results:**

This review includes twenty studies, comprising twelve clinical trials, two case studies, and six animal studies. The most commonly reported actions of oxymel include positive effects on the cardiovascular system, as well as antioxidant and anti-inflammatory properties. Furthermore, compound oxymel formulations have demonstrated additional benefits depending on the inclusion of specific medicinal herbs.

**Conclusion:**

Based on our findings, oxymel appears to be a valuable functional food for healthy individuals and a potentially effective and safe treatment option for managing certain diseases such as asthma, obesity, and type 2 diabetes. However, further clinical trials with larger sample sizes and longer durations are needed to fully elucidate the potential side effects and benefits of both simple and compound oxymels in various disease states.

## 1 Introduction

Functional foods are a category of foods that not only provide nutritional value but also have positive effects on health [1]. This concept aligns with the oriental philosophy that “Medicine and food have a common origin” [2]. The term “functional food” was first introduced in the 1980s in Japan as a response to rising healthcare costs. Subsequently, the Ministry of Health and Welfare implemented a system to approve specific foods as functional foods based on their documented health benefits [1].

Although the concept of functional foods is often regarded as a new and emerging field, it has been described in historical texts and has a long history of use in various ethnic cultures and traditional medical literature worldwide. Examples include ancient Indian Vedic texts and traditional Chinese medical literature [[2], [3], [4]]. In Iranian culture and traditional medicine, known as Persian medicine (PM), numerous functional foods have been utilized for thousands of years and continue to be consumed today [[5], [6], [7]].

In PM, foods and drinks (i.e. nutrition) are considered one of *the six essential principles* for maintaining good health. These principles encompass “nutrition, exercise, sleep and wakefulness, excretion of body wastes and retention of necessary materials, air and climate, as well as psychological factors”. Based on PM teachings, the aforementioned six principles are the most important medical rules for a healthy lifestyle [[8], [9], [10]].

Moreover, food therapy is one of the primary treatment modalities in PM and is considered the first-line approach. PM scholars emphasized that the initial step in treating diseases should involve dietary modifications rather than drug prescriptions. They believed that the potential side effects of food are much lower compared to those of drugs, and it is preferable for physicians to treat a disease through dietary modifications rather than resorting to drug prescriptions [8]. In several prominent PM texts, including “Canon of Medicine” (*Al-Qanun Fi al-Teb*) by Avicenna (980–1037 AD) [11], “Comprehensive Book on Medicine” (*Al-Hawi Fi al-Teb*) by Rhazes (865-925 AD) [12], and “The Perfect Book of the Art of Medicine” (*Kamel al-Sana'a al-Tebbiya*) by Haly Abbas (949-982 AD) [13], there are numerous food recipes recommended for various diseases.

One of the most commonly prescribed food-drug preparations for a wide range of ailments is oxymel, which has a historical usage dating back to the time of Hippocrates [[14], [15], [16]]. The term “oxymel” is derived from the Greek words “oxymeli,” meaning acid and honey. Not only is the Greek word “oxymeli” mentioned in PM sources, but physicians such as Hippocrates and Galen have also been extensively referenced in PM books for their discussions on the therapeutic properties of oxymel [[17], [18], [19]].

In ancient Persia, this beverage was known as *serkangabin*, derived from the combination of two words, *serkeh* (vinegar) and *angabin* (honey), as vinegar, honey/sugar, and water are the main ingredients of oxymel [14]. As its use expanded to neighboring cultures, the term underwent phonetic changes. For example, it became *sekanjabin* in Arabic, which is also used in modern Persian. In Turkey, it is written as *sirkencubin*, while in India, it is referred to as *shikanji* or *sikanjabeen* [[20], [21], [22]].

**Fig. 1:**
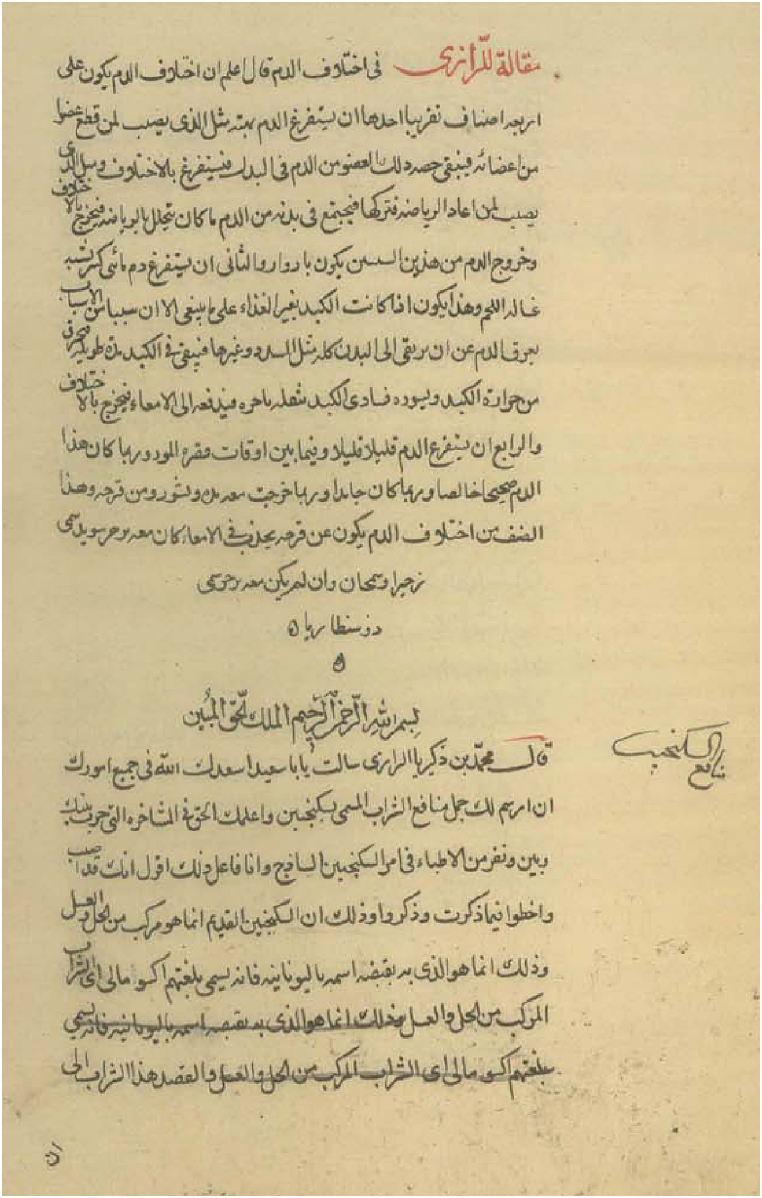
The first page of “*Resale fi Manafe- Sekanjabin*” (treatise on oxymel's benefits); kept in the Library of the Iranian Parliament; record number: 903795–1010123.

Various PM textbooks have detailed different types of oxymel, including their preparation methods, indications, benefits, and side effects. There is even a dedicated treatise solely focused on oxymel, although there is some debate regarding whether it was authored by Avicenna or Rhazes (Fig. 1) [19,23]. The formulations and indications of oxymel use in Unani Medicine in India share many similarities with PM and are considered comparable to PM [[24], [25], [26]].

In the case of individuals with a hot temperament or during hot seasons and climates, a simple oxymel is typically prepared using sugar, while honey is used in other cases [19]. Compound oxymels involve the addition of various medicinal plant seeds, roots, leaves, and fruit extracts [18,19,27]. Among the widely used compound oxymels is squill oxymel, which has gained recognition in the West and continues to be used today [16,28]. PM texts described over 1200 types of oxymels [14,15]. Furthermore, oxymels are utilized in the preparation of certain drugs, such as whey formulations [29,30].

Medicinal oxymels find application in a broad range of disorders, including liver, spleen, gastrointestinal, and respiratory diseases [14,15,18]. Oxymels have been prescribed not only for chronic conditions but have also been frequently employed in the management of critically ill patients with acute diseases [31]. For instance, the Canon of Medicine mentions the use of oxymel in the treatment of febrile illnesses and critically ill patients who are on the verge of the critical stage of the disease [18,32,33].

In recent years, the food industry has seen a surge in studies focusing on oxymel [20,21,34,35]. These studies have explored various aspects of oxymel, including its pharmacological properties [27,29,36]. Additionally, clinical trials, animal studies, and case reports have been conducted to assess its clinical effects across a wide range of diseases. Despite this growing body of research, to the best of our knowledge, no article has provided a comprehensive review of the existing studies. Therefore, the aim of this study is to fill this gap by conducting a thorough review of both preclinical and clinical studies on oxymel.

## 2 Methods

The Embase, MEDLINE, Web of Science Core Collection, Scopus, and Google Scholar databases were searched from their inception to July 2023 to identify all articles on oxymel. Keywords used included oxymel, *sekanjabin*, *serkangabin*, *sikanjabin*, *sikanjabeen*, *sikanjbeen*, *sirkencubin*, *shikanji*, and honey vinegar. The search was conducted without any restrictions to capture all relevant studies. Two independent researchers (N.S.D and M.H.H.) reviewed the titles and abstracts of the results to exclude irrelevant articles and papers from unrelated fields (such as photography). After obtaining the full texts of the included studies, the reference sections were manually examined to identify any additional new articles.

Inclusion criteria encompassed studies addressing preclinical or clinical aspects of oxymel, including clinical trials, animal studies, case studies, and case reports, regardless of the oxymel formulation and dosage. Historical articles, reviews, and studies related solely to the food industry and formulation were excluded. Additionally, articles in which oxymel was an ingredient of a formula were included, while those in which oxymel was used as a starter were excluded.

**Fig. 2:**
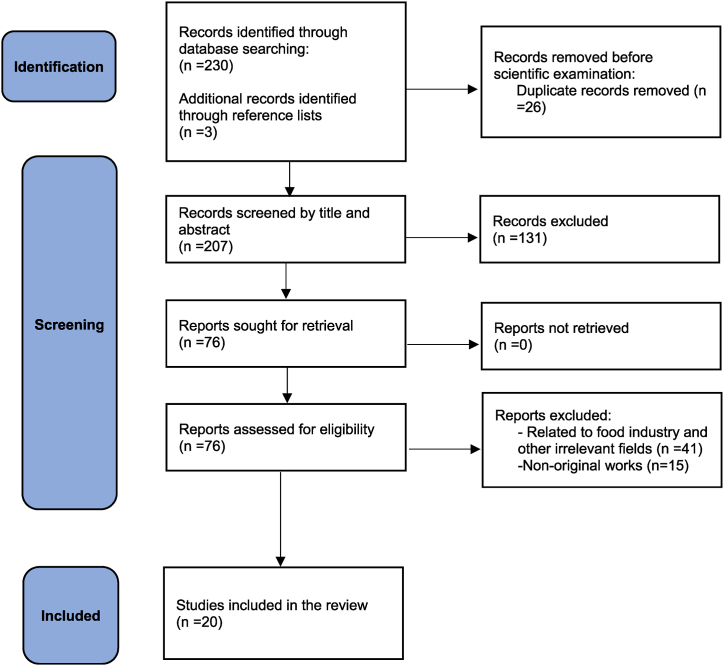
PRISMA flow chart of the study.

Upon reviewing the full texts of the remaining articles, the two researchers extracted the following information: first author's name, publication year, country, study design, sample population (sample size, type of disease), the mean age of participants, gender, oxymel formulation, duration and type of intervention, control groups, outcome measures, results, and adverse events. Fig. 2 depicts the flow chart of our review.

## 3 Results

Twenty interventional studies were conducted on oxymel, comprising six animal studies, two case series, and twelve clinical trials. Seventeen of these studies, including nine clinical trials, were conducted in Iran, with the remaining three articles from India, Turkey, and Indonesia.

The most commonly investigated diseases were type 2 diabetes mellitus (T2DM) and obesity, with three studies on T2DM [[37], [38], [39]] and three on obesity [37,40,41]. Asthma was the next most studied disease with two studies [42,43] and one study focused on COPD [44]. Among studies on healthy volunteers, two studies investigated the effect of oxymel on blood pressure [[45], [46], [47]].

The most frequently used type of oxymel was simple oxymel, followed by oxymel containing Shirazi thyme (*Zataria multiflora* Boiss.) and squill (*Drimia Maritima* (L.) Stearn) with two studies. Other types of oxymel containing common barberry (*Berberis vulgaris*), caper bush (*Capparis spinosa*), and lemon (*Citrus lemon* L.) were each investigated in one study. Generally, compound oxymels containing plants of the mint family (Lamiaceae), including mint (Mentha), peppermint (*Mentha piperita*), Shirazi thyme (*Zataria multiflora* Boiss.), and purple basil (*Ocimum bacilicum*) were the second most frequently used oxymels, following simple oxymel. The essential ingredients of simple oxymel include a sweet component (such as sugar, honey, dextrose, and lactulose), vinegar, and water. Sugar was the most commonly used sweet component in the reviewed studies.

Most studies did not report any side effects of oxymel. However, five articles mentioned complications including hypersensitivity reactions, hyperglycemia, gastrointestinal complications, hypothyroidism states, and headache in only a few participants [37,42,[46], [47], [48]], the details of which are provided in Table 1. Overall, none of the types of oxymels studied had serious or severe adverse effects on humans or animals.

**Table 1:** Human interventional studies on oxymel.

Study	Study design	Sample population	Mean age (yrs.)/Gender	Oxymel formulation	Intervention	Control	Outcome measures	Results/main conclusion	Adverse events
Taheri et al., 2023 (48) Iran	triple-blind RCT	N = 88 (43 I, 45 C) Patients with knee osteoarthritis	I: 54.13 ± 7.53 C: 55.08 ± 7.12/70 F, 81 M	Based on the company's documents, the squill oxymel syrup was manufactured by boiling squill vinegar extract (33.3 %) and honey (66.7 %)	squill oxymel syrup (10 ml each morning on empty stomach) for 8 weeks	brown sugar syrup (50 % w/w) was used as a placebo	The Knee injury and Osteoarthritis Outcome Score and VAS were considered as the main outcomes. Laboratory tests including AST, ALT, BUN, Cr, WBC, ESR, and CRP, specific tests i.e., IL6 and SOD as the secondary outcomes.	A significant positive effect of treatment was observed on the outcome of knee pain and daily activity of KOOS after Cessation of treatment. No significant effect on VAS. After 4 weeks of cessation of treatment, the positive effect of the squill oxymel on the treatment group continued in some of the subscales of KOOS, including symptoms, knee pain and daily activities, but stopped in the placebo group. Both clinically and statistically significant improvement was observed after cessation of oxymel	Intervention group: nausea (n = 3), constipation (n = 2), exacerbation of knee stiffness (n = 1), headache & dizziness (n = 1). Placebo: a woman who had a history of allergy to cold water, experienced redness, itching and inflammation of the palms, nausea and other GI problems (n = 2), loss of appetite (n = 1). No serious adverse effect
Mohammadi–Araghi et al., 2022 (44) Iran	triple-blind RCT	N = 42 (21 I, 21C) COPD patients with moderate disease (Stage IIA)	I:60.00 ± 8.99 C:61.10 ± 7.78 /4 F, 38 M	The squill rhizomes were cleaned and washed, broken into pieces, boiled in vinegar, and then honey was added after the bulbs softened.	Squill-oxymel syrup; twice a day, once in the morning while fasting and again before bedtime, at a dosage of 10 ml each time. It was added to the patients' regular therapies.	Placebo syrup (was made from honey cooked in water); twice a day, once in the morning while fasting and again before bedtime, at a dosage of 10 ml each time. It was added to the patients' regular therapies.	Distance in the 6MWT, O_2_ saturation at the start of the 6MWT, O_2_ saturation at the end of the 6MWT, and St. George's Respiratory Questionnaire scores	The intervention had a significant effect on the distance in the 6MWT, O_2_ saturation at the end of the 6MWT, and St. George's symptom scores. Other St. George's scores were not statistically changed in the two groups.	No side effects were reported
Mahdavi-Roshan et al., 2021 (40) Iran	double-blind RCT	N = 96 (49 I, 47 C) Overweight and obese healthy people	I: 35.29 ± 7.27 C: 35.21 ± 7.87 /63 F, 33 M	Grape vinegar, sugar, and water in proportions of 1:2:4 was heated for 1 h.	Dietary recommendations for weight control + 30 ml oxymel per day, which was added to 250 ml water, as an evening meal, for 30 days.	Dietary recommendations for weight control +250 ml water bottles without oxymel	Weight, BMI, WC, HC, WHR, SBP, DBP, FBS, TG, TC, HDL, LDL	The intervention had a significant effect on the serum cholesterol and body weight, but did not affect other anthropometric and biochemical indicators.	No side effects were reported.
Naghibi et al., 2021 (55) Iran	Quasi-experimental study	N = 25 (13 PSC, 12 PBC) Patients with refractory PSC and PBC	PSC: 34.15 ± 10.58 PBC: 43 ± 15.23 /17 F, 8 M	Water, dextrose, grape vinegar, and rose water in proportions of 8:8:1.5:0.5 were boiled. After cooling the oxymel, *Berberis vulgaris* fruit extract (1.1 g per 15 ml) was added to prepare the *Berberis vulgaris* oxymel (BO).	0.5 ml/kg/day BO twice daily for 3 months along with ursodeoxycholic acid	No control group	ALP, AST, ALT, DB, TB, GGT, PT, INR. QOL (PBC-40 questionnaire)	BO significantly attenuated ALP, AST, ALT, GGT, TB, DB, PT and INR, and QOL significantly improved.	No side effects were reported.
Abolghasemi et al., 2020 (37) Iran	Randomized controlled triple-blind clinical trial	N = 92 (32 I_1_, 31 I_2_, 29 C) Overweight participants	I_1_: 35.56 ± 9.89 I_2_: 39.65 ± 9.37 C: 40.28 ± 9.33 /70 F, 22 M	Sugar, vinegar, and distilled water in proportions of 3:1:1 was boiled. For every 20 ml of syrup, 1.5 g or 3 g *Zataria multiflora* Boiss. (ZM) was added for group I_1_ or group I_2_ respectively, and decoct for 15 min.	**Group I**_**1**_**:** 0.75 g ZM in 10 ml oxymel; **Group I**_**2**_**:** 1.5 g ZM in 10 ml oxymel; for 12 weeks, each was mixed with 100 ml water and administered twice daily before breakfast and 1 h before bedtime.	Ten ml of oxymel	BMI, WC, HC, WHR, FBS, Insulin, HOMA-IR, TG, TC, LDL, HDL	Oxymel and Zataria oxymel improved metabolic parameters including reducing HOMA index, insulin resistance, WC, HC, and WHR, without changing TG, TC, and BMI.	Hypersensitivity reaction (one patient in each group), GI problem (one patient in I_2_ group), hyperglycemia (one patient in C group), and hypothyroidism (one patient in C group).
Yikmiş et al., 2020 (45) Turkey	Uncontrolled before-after study	N = 10 Healthy volunteers	20.20 ± 0.78 /6 F,4 M	Oxymel syrup: 14 g honey + 6 ml vinegar + 200 ml sterile water were homogenized by homogenizer for 2 min. Purple basil oxymel syrup: 14 g honey +6 ml vinegar +200 ml purple basil tea (7.5 g purple basil/L) were homogenized by homogenizer for 2 min.	In the first stage, BP measurements were performed 2 h after the breakfast. Then, BP measurements were performed again 30 min after drinking 400 ml oxymel syrup. In the second stage, BP measurements were made before and after drinking 400 ml purple basil oxymel syrup, in the same way.	No control group	SBP, DBP	Oxymel syrup had significant effect on DBP. Purple basil oxymel syrup had no significant effect on participants' SBP and DBP.	No side effects were reported.
Vahid et al., 2019 (38) Iran	triple-blind RCT	N = 30 (10 I_1_, 10 I_2_, 10 C) Poorly controlled type 2 diabetic patients with metabolic syndrome	I_1:_ 55 ± 4 I_2:_ 55 ± 6.6 C: 58 ± 3.6 /19 F, 11 M	Simple oxymel: water, grape vinegar, and lactulose in proportions of 5:1:2 was boiled. *Capparis spinosa* oxymel (CO): hydroalcoholic extract of *Capparis spinosa* fruit + simple oxymel (1 g per 10 ml)	Ten ml of simple oxymel, or CO thrice daily for 3 months + conventional therapy with hypolipidemic, antihyperlipidemic, and antihypertensive drugs	Ten ml of diluted lactulose in distilled water + conventional therapy with hypolipidemic, antihyperlipidemic, and antihypertensive drugs	Weight, BMI, WC, SBP, DBP, FBS, PPBS, HbA1c, TG, TC, LDL, HDL	CO significantly decreased the weight and BMI, and inhibited the progression of hyperglycemia. There was not a significant difference between placebo and intervention groups regarding HbA1c and lipid profile.	No side effects were reported.
Anjum et al., 2018 (54) India	Uncontrolled before-after study	N = 30 Pregnant women with mild and moderate nausea and vomiting	22.03 /30 F	Lemon oxymel containing lemon Juice, acetum vinegar, sugar and citric acid.	Lemon oxymel 25 ml twice daily orally for 2 weeks	No control group	Assessment of severity of nausea and vomiting by PUQE, assessment in improvement of QOL by NVPQOL questionnaire	PUQE and NVPQOL scores significantly decreased on day 7, continued through day 14 and after treatment. 86.6 % patients achieved complete relief from NVP at the end of treatment period.	No side effects were reported.
Nejatbakhsh et al., 2017 (42) Iran	triple-blind RCT	N = 54 (18 I_1_, 18 I_2_, 18 C) Patients with moderate to severe persistent asthma	I_1_: 49.78 ± 9.2 I_2_: 40 ± 9.74 C: 48.5 ± 16.53 /30 F, 24 M	Squill oxymel (SO): small pieces of squill bulbs boiled in vinegar and then honey was added. Simple oxymel was prepared with a base of oxymel syrup without squill.	I_1_: 10 ml simple honey oxymel I_2_: 10 ml SO. Each one: twice daily for 6 weeks, as adjuvant	Ten ml of honey boiled in water, twice daily for 6 weeks	Spirometry: FEV1 liter, FEV1%, FEV1/FVC%, MEF 25–75 %, actual PEF. Body plethysmography: actual TLC, RV, RV/TLC, RAW total, RAW ex, RAW in, TGV, TGV/TLC, IC, sRaw. Health-related QOL by SGRQ	FEV1 liter, FEV1%, FEV1/FVC%, and MEF 25–75 % were significantly increased during the intervention in SO group than in the other groups (p < 0.001). The improvement of plethysmographic parameters showed no significant difference between the study groups. The SGRQ total score and its sub-scores significantly improved after intervention in both I_1_ and I_2_ groups (p < 0.001).	Two patients in SO group and 3 patients in simple oxymel group developed nausea and vomiting, respectively.
Derakhshandeh-Rishehri et al., 2015 (46) Iran	open-label RCT	N = 61 (30 I, 31 C) Healthy volunteers	I: 28.3 ± 4.3 C: 31.6 ± 6.9 /39 F, 22 M	One kg honey + 1500 ml water heated (few minutes) and let to condense. Then vinegar (300 g) was added.	Normal Diet +2 tablespoons oxymel (21.7 g) with 250 ml water in evening snack daily for 4 weeks	Normal Diet	Weight, BMI, WC, HC, WHR, Subcutaneous fat thickness, SBP, DBP	No significant effect of oxymel on BP and anthropometric indices	Stomach ache, nausea and headache after 15 days of intervention in one participant
Derakhshandeh-Rishehri et al., 2014 (47) Iran	open-label RCT	N = 61 (30 I, 31 C) Healthy volunteers+/Healthy volunteers	I: 28.33 ± 4.32 C: 31.61 ± 6.86 /39 F, 22 M	One kg honey + 1500 ml water heated (few minutes). Few mint branches added, then syrup let to condense. Then vinegar (300 g) was added.	Normal Diet (based on healthy food pyramid) + 2 tablespoons oxymel (21.66 g) plus water (250 ml) in early evening or mid-morning snack daily (4 weeks)	Normal Diet (based on healthy food pyramid)	FBS, Insulin, HOMA-IR, TC, TG, HDL, LDL	No significant effect of oxymel on lipid profile and FBS except for HDL. TC decreased, fasting insulin significantly increased, and HDL significantly decreased in the intervention group.	Stomach ache, nausea and headache after 15 days of intervention in one participant
Firoozabadi et al., 2014 (56) Iran	open-label RCT	N = 60 (30 I, 30 C) Patients with migraine with or without aura	I: 31.7 ± 7.6 C: 32.6 ± 12.7 /41 F, 19 M	Honey + water + vinegar + peppermint water was boiled. A cup of this syrup was mixed with 4–5 cups water. Each 200 ml of oxymel syrup consists of 15 ml honey, 15 ml distilled peppermint water and 7 ml grape vinegar.	Cupping therapy plus oxymel for 6 months: 200 ml of oxymel syrup every night for 60 days from the first appearance.	Conventional medical treatment (6 months): 1. Ergotamine tartrate USP 2 mg in the beginning of attack and every 30 min if needed for pain control (max dose: 6 mg). 2. Sumatriptan succinate USP 50 mg tablet (when no medication response or ergotamine intolerance) early in the attack and repeated every 2 h if needed (max dose: 200 mg). 3. Nortriptyline or propranolol as prophylactic treatments	Severity of headache by VAS, frequency of attacks in a week, duration of attacks	No significant difference between conventional treatment and cupping + oxymel therapy, regarding severity, frequency and duration of headaches were reported.	Not mentioned.

AST: aspartate transaminase; ALT: alanine transaminase; ALP: alkaline phosphatase; BMI: body mass index; BP: blood pressure; BSA: body surface area; COPD: chronic obstructive pulmonary disease; C: control group; DBP: diastolic blood pressure; DB: direct bilirubin; DLQI: dermatology life quality index; FBS: fasting blood sugar; F: female; FEV1: forced expiratory volume in first second; FVC: forced vital capacity; GGT: gamma-glutamyl transferase; GI: gastrointestinal; HbA1C: glycated hemoglobin; HC: hip circumference; HDL: high density lipoprotein; HOMA IR: homeostasis model assessment of insulin resistance; IC: inspiratory capacity; I: intervention group; I1: intervention Group 1; I2: intervention Group 2; INR: international normalized ratio; LDL: low density lipoprotein; M: male; NVP: nausea and vomiting in pregnancy; NVPQOL: nausea and vomiting in pregnancy-specific quality of life questionnaire; PASI: psoriasis area and severity index; PBC: primary biliary cholangitis; PEF: peak expiratory flow; PPBS: postprandial blood sugar; PSC: primary sclerosing cholangitis; PT: prothrombin time; PUQE: pregnancy unique quantification of emesis; QOL: quality of life; RCT: randomized controlled trial; RV: residual volume; SBP: systolic blood pressure; sRaw: specific airway resistance; SGRQ: St. George's respiratory questionnaire; SOD: superoxide dismutase; TB: total bilirubin; TG: triglyceride; TC: total cholesterol; TLC: total lung capacity; TGV: thoracic gas volume; VAS: visual analog scale; WC: waist circumference; WHR: waist to hip circumference ratio; MEF25-75 %: forced expiratory flow between 25 % and 75 %; RAW (ex, in): airway resistance (inspiratory, expiratory); 6MWT: 6-min walking test.

### 3.1 Animal studies

The six animal studies assessed the effect of oxymel on clinically relevant animal models, including the asthmatic mice model, the diet-induced obesity model in rats, the diet-induced dyslipidemia model in rats, the streptozotocin-induced diabetic rats, and the pentylenetetrazole-induced seizure model in mice [39,41,43,[49], [50], [51]]. Four studies were performed on rats, and two were performed on mice. Sugar and honey were used as the sweet component in four and one studies, respectively. Moreover, four studies investigated simple oxymel, and one assessed Shirazi thyme oxymel. A summary of animal studies is provided in Table 2.

**Table 2:** Animal studies of oxymel.

Study	Sample population	Age, Weight, Gender	Oxymel formulation	Intervention	Control	Outcome measures	Results/main conclusion	Adverse events
Abolmaali et al., 2022 (51) Iran	N = 39 (6 equal groups) NMRI mice, PTZ-induced seizure	Age: not reported 30±4 g, M	Containing squill bulb, vinegar, and honey. Details were not reported.	The positive control group (group 2) received diazepam (1mg/kg, IP) 15min before the administration of PTZ Groups 3–6 mice were given different doses of *Drimia Maritima* oxymel (50, 100, 200, 400 mg/kg) by oral gavage, 15min before the injection of chemo-convulsant to animals.	The control group received normal saline (0.9 %, IP) as group 1.	The period of time before the convulsion onset (latency), duration of clonic0 convulsion and the percentage of animal mortality and protection	A prolonged time of onset of seizure was observed with higher doses of 100 and 200 mg/kg (P˂0.05). Moreover, squill oxymel at the doses of 100, 200 and 400 mg/kg decreased the duration of seizure in comparison to the normal saline control group (P˂0.05). Squill oxymel showed its impact on reducing the mortality rate of animals in a dose-dependent manner.	No side effects were reported.
Faryabi et al., 2022 (43) Iran	N = 42 (6 equal groups) BALB/c mice, Asthma	6–8 weeks, 15–20 g, M	100 ml vinegar (pH: 3.37) + 200 ml water were heated. Before the solution came to a boil, 300 g sugar was added and stirred. After the solution came to a boil, the heat was turned down immediately. For gavage, 0.2 ml of the syrup (pH: 3.52) was dissolved in 0.8 ml of water and then cooled down to 45 °C after being heated again up to 100 °C.	1. Oxymel group was gavaged with 0.1 ml warm (45 °C) oxymel. 2. Oxymel + sauna (OS) group received warm oxymel gavage followed by placement in the sauna chamber. Treatment duration: 10 days	1. Sauna group: Placed in a closed warm dry incubator (sauna chamber) of 37 °C for 30 min. 2. Budesonide group: Treated with 1 mg nebulized budesonide by administering 200 μg/puff every 5 min for 25 min. Treatment duration: 10 days. 3,4. Asthma and control groups: Did not receive any treatment.	Gene expression of IL-4, IL-5, MUC5AC, IFN-γ; Histological analysis of lung tissue (Perivascular inflammation, peribronchial inflammation, goblet cell hyperplasia, mucus hypersecretion).	OS compared to the untreated asthmatic group: Reduction in IL-4, IL-5, and MUC5AC gene expression, decrease in peribronchial and perivascular inflammation, goblet cell hyperplasia, and mucus hypersecretion, increase in IFN-γ gene expression and IFN-γ/IL-4 ratio. OS similar to budesonide: Decreased IL-5 and MUC5AC gene expression levels. OS action was more effective than budesonide in: Increase in IFN-γ gene expression level. Oxymel alone, compared with the untreated asthma group, only: Decreased IL-4 gene expression, mucus hypersecretion, perivascular and peribronchial inflammation.	No side effects were reported.
Nimrouzi et al., 2020 (41) Iran	N = 80 (11 groups) Sprague-Dawley rats, Obesity	6–8 weeks, 220 ± 15 g, M	Simple oxymel: 1 L grape vinegar (acidity: 5.2) + 3000 g sugar + 1 L distilled water boiled up to its consistency. Thyme oxymel (TO): 300 mg (for the 4th, 7th and 10th groups) or 500 mg of thyme powder (for 5th, 8th and 11th groups) was added to 20 ml simple oxymel. The solution was boiled for 10 min and then filtered.	The 3rd, 4rth and 5th groups received 500 mg/kg/BW of oxymel, 300 mg/kg/BW of TO, and 500 mg/kg/BW of TO, respectively every day for 12 weeks. The 6th, 7th and 8th groups simultaneously received HFFD 500 mg/kg/BW of oxymel, 300 mg/kg/BW of TO, 500 mg/kg/BW of TO, respectively every day for 12 weeks. The 9th, 10th and 11th groups where obesity was induced by HFFD for 12 weeks and then treated with 300 mg/kg/BW of oxymel, 300 mg/kg/BW of TO, and 500 mg/kg/BW of TO, respectively every day for 12 weeks.	1. First group: as a control group received 1 ml/d distilled water. 2. Second group: Obesity was induced by HFFD for 12 weeks.	AST, ALT, TC, TG, LDL, HDL, FBS, insulin, HOMA-IR, leptin, SOD, GPx, CAT, MDA, TNF-α, zinc, manganese, copper, iron; genes expression of SREBP-1c, CPT-1, Nrf-2 and NF-κB; Histopathological examination of the liver.	Pretreatment and treatment with TO and oxymel in obese rats significantly ameliorated the level of oxidative biomarkers, TNF-α, lipid profiles, leptin, liver enzymes, insulin and the levels of some trace elements and bring some of them back to normal (p < 0.05), and also significantly ameliorated up-regulation of the expression of SREBP-1c and NF-κB and down-regulation of CPT-1 and Nrf-2 expression (p < 0.05). In conclusion, oxymel or TO can alleviate HFFD-induced obesity through improving inflammation, oxidative stress, lipid metabolism, homeostasis of some trace elements, and weight regulating hormones.	No side effects were reported.
Sarbaz Hoseini et al., 2019 (39) Iran	N = 5 Wistar rats, Type 2 diabetes	4 weeks, 150–200 g, M	One kg sugar + 500 ml warm water was boiled. Then 300 g vinegar was added and boiled again. For gavage, 0.2 ml oxymel was mixed with 0.8 ml water which reached 100 °C, then cooled to 50 °C.	Rats were gavaged with 1 ml oxymel 5 days a week and then placed in a sauna chamber at 37 °C for 30 min. The treatment performed for 4 weeks.	No control group	Weight, FBS.	Rats' weight significantly increased after the treatment period (P = 0.022), and the mean FBS decreased (P = 0.049).	No side effects were reported.
Sarbaz Hoseini et al., 2019 (49) Iran	N = 64 (8 equal groups) Wistar rats, Type 2 diabetes	8 weeks, 180–220 g, M	Sugar (1 kg) boiled with water (500 ml) then vinegar (300 g) added. For gavage, 0.2 ml of oxymel dissolved in water (0.8 ml) first boiled to 100 °C, then cooled to 50 °C.	1. Diabetic group: Treated by gavage with oxymel (1 ml) (5-days a week for 8 weeks) (OX). 2-4. Diabetic groups: Treated by both sauna and oxymel and for one (OS1d), three (OS3d), and five days (OS5d2m) a week for 8 weeks. 5. Diabetic group: Treated by both oxymel and sauna (5-days per week for only 4 weeks then no intervention for the next 4 wks (OS5d1m). All diabetic groups had a high-fat diet	1. A healthy control group (Nl); normal diet throughout 8 wk study + first day single dose of citrate buffer (as streptozotocin solvent 2. An untreated diabetic group (D) 3. A sauna treated diabetic group (5 days a week) (Sauna).	Weight, FBS, HbA1C, insulin, HOMA-IR, TG, TC, HDL, LDL; histological assessment of pancreas, liver, kidney and heart.	1.Langerhans Islands count: significant increase in OS3d group (P = 0.05) and OS5d2m group (P = 0.008) in comparison to untreated D group 2. Organization of cells became nearly normal 3. Glucose levels & Serum insulin not change. 4. Despite high-fat diet, histological findings and lipid profile was not compatible with insulin resistance & fatty deposition context. 5. OS significantly induced Langerhans Island regeneration, but its effect on beta-cell functions and insulin resistance yet to be proven.	No side effects were reported.
Lucia et al., 2017 (50) Indonesia	N = 30 (3 equal groups) Wistar rats, Hyperlipidemia	2–3 months, 150–250 g, M	Not mentioned	The test group was given fried oil (4 ml/kg/BW), pork oil (5 ml/kg/BW), and oxymel (10 ml/kg/BW) orally, for 14 days.	1. The negative control group was given demineralized water. 2. The positive control group was given fried oil (4 ml/kg/BW) and pork oil (5 ml/kg/BW) orally, for 14 days.	LDL, HDL, TG, TC.	The test group treated with oxymel had a significantly lower lipid profile than the positive control group (P < 0.05).	No side effects were reported.

ALT: alanine transaminase; AST: aspartate transaminase; CAT: catalase; CPT-1: carnitine palmitoyl transferase I; GPx: glutathione peroxidase; HbA1C: glycated hemoglobin; FBS: fasting blood sugar; HDL: high-density lipoprotein; HFFD: high-fructose and-fat diet; HOMA-IR: homeostasis model assessment of insulin resistance; IFN-γ: interferon-gamma; IL: interleukin; LDL: low-density lipoprotein; M: male; MDA: malondialdehyde; MUC5AC: Mucin 5 Subtypes A And C; NF-κB: nuclear factor kappa B; Nrf-2: nuclear factor erythroid 2-related factor 2; NMRI: Naval Medical Research Institute; PTZ: pentylenetetrazole; SREBP-1c: sterol regulatory element-binding transcription factor 1; SOD: superoxide dismutase; TC: total cholesterol; TG: triglyceride; TNF-α: tumor necrosis factor-alpha.

### 3.2 Case series

One study examined the effect of oxymel on two middle-aged men with prostate pain syndrome [52]. Conventional medications had not relieved their symptoms, which was the reason they had referred to a PM clinic. The oxymel used in this study was of simple sugar type and administered in a dose of 15 ml, 45 min after lunch and dinner for four weeks. The results indicated a reduction in pelvic pain and urinary symptoms and an improvement in the quality of life of both patients. No side effects were reported. Another study assessed the effect of oxymel alone or in combination with other PM recommendations in the treatment of pediatric constipation in six children, including four girls and two boys, for one month [53].

The children aged 2–10 years and had not responded to conventional therapies for constipation. Simple oxymel was a component of the prescribed regimen for four participants, while two children received oxymel alone. The oxymel formula and dosage were not mentioned in the article. Based on the results, constipation and associated symptoms improved in the two participants who used oxymel alone, and no side effects were observed.

### 3.3 Clinical trials

The efficacy and safety of oxymel have been evaluated in twelve clinical studies on various diseases. Investigated diseases included obesity [37,40], T2DM [38], nausea and vomiting during pregnancy [54], asthma [42], chronic obstructive pulmonary disease (COPD) [44], refractory primary sclerosing cholangitis (PSC) and primary biliary cholangitis (PBC) [55], migraine headaches [56], and knee osteoarthritis [48]. Overweight and obesity have been studied more than other diseases, with two articles [37,40]. Furthermore, three studies were designed and conducted on healthy volunteers [[45], [46], [47]].

The sample size ranged between ten and ninety-six participants. Except for one study on pregnant women, other reviewed research included both genders. The duration of interventions varied from one day (on healthy volunteers) to six months (on migraine patients).

Different oxymel formulations were used with varied daily dosing from one to three times a day. The sweet component used to prepare oxymel was honey in five studies, sugar in three, and dextrose and lactulose in one study. A summary of clinical trials is demonstrated in Table 1.

## 4 Discussion

Considering that the majority of studies on oxymel have been conducted in Iran using preparation methods based on PM sources, the preparation method of this food-drug substance is naturally similar in Iranian articles. While some studies have explicitly stated that oxymel was prepared by heating and cooking raw materials, this is not the case for all reviewed articles. For instance, in one study, oxymel was prepared by mixing raw materials with a homogenizer without boiling or cooking [45].

Out of the twenty articles reviewed, fourteen examined the effects of simple oxymel. Some studies investigated oxymel as a single intervention, while others compared or combined it with other interventions such as compound oxymel, cupping, and thermal therapy. Therefore, the analysis of the mechanism of action will differ in each of the above cases.

The mechanism of action of oxymel in the aforementioned studies can be discussed on different levels. Firstly, by considering the essential ingredients of simple oxymel (the sour and sweet components) and their synergistic effects. Secondly, by considering the herbal medicines included in the formulations. Finally, by considering the adjuvant therapies used alongside oxymel.

### 4.1 Pharmacological effects of vinegar

Regarding vinegar, there are reports of its beneficial effects on improving the risk factors for cardiovascular disease and diabetes [46]. Reduction of serum leptin and visceral fat, improvement of lipid profile, weight loss [37], anti-inflammatory properties, and antioxidant effects [55] are all mentioned in the literature.

As the main component of vinegar, acetic acid is deemed responsible for the various effects of vinegar, such as reduction of fat accumulation [37], weight loss [46], antihypertensive properties [21,38,42,46,54], effects on blood glucose metabolism, reduction of insulin resistance and insulin levels (with the possible mechanism of reducing hepatic and musculoskeletal glycolysis, inhibition of alpha-amylase activity, and decrease in carbohydrate digestion) [37]. The acetate in vinegar stimulates fatty acid oxidation and inhibits lipogenesis, resulting in potentially positive effects on the lipid profile.

Grape vinegar is a rich source of antioxidants and phenolic compounds [46]. The phenolic compounds and acetic acid in grape vinegar inhibit weight gain and accumulation of body fat [41]. Its polyphenolic compounds also improve insulin sensitivity and release [47], suppress adipocyte differentiation, and inhibit lipid accumulation in the liver by downregulating the expression of enzymes involved in hepatic triglyceride synthesis [41].

### 4.2 Pharmacological effects of honey

Regarding honey, beneficial effects on modifiable cardiovascular and diabetes risk factors are reported in the literature [46,47]. Mechanisms of weight loss from honey consumption include a rich content in fructose and oligosaccharides, modulation of appetite-regulating hormones, including leptin and ghrelin, reduction of protein digestion and absorption, increased fecal nitrogen, increased levels of antioxidant compounds in the blood, and modulation of oxidative stress in tissues [46].

Copper and zinc in honey play a role in modifying insulin and glucose metabolism [46,47]. Antioxidants lead to weight loss and improvement of the lipid profile [46,47]. Furthermore, honey has proven to reduce prostaglandin levels and increase nitric oxide. Prostaglandin E2 is an essential inhibitor of glucose-induced insulin secretion [47].

### 4.3 Pharmacological effects of oxymel (a combination of vinegar and honey)

Vinegar or honey alone can each reduce the risk factors for cardiovascular disease or diabetes, and their combination as oxymel can offer the benefits of both foods simultaneously [46]. Moreover, the synergistic interaction of these two nutrients in oxymel processing can lead to new and different effects from consuming each one separately [46,47]. Phytochemical analysis of oxymel in one study showed that it contains high levels of phenolic compounds and has a high antioxidant capacity [41].

### 4.4 Pharmacological effects of compound oxymels

Regarding compound oxymels, various action mechanisms may be discussed, depending on the substance added to the base oxymel formulation. An example is emphasizing the beneficial effects of *Berberis vulgaris* fruit in liver disease, pointing out the antioxidant and anti-inflammatory effects of both *Berberis vulgaris* and grape vinegar, and mentioning that their combination reinforces their therapeutic effects [55].

Vahid et al. ascribed the anti-hyperglycemic effects of *Capparis spinosa* to its phenolic compounds [38]. The mechanisms were stated to reduce the intestinal absorption of carbohydrates, enhance glucose uptake in tissues, inhibit glucose production in the liver, and protect and regenerate the pancreatic islets.

Nejatbakhsh et al. attributed the positive effects of squill oxymel in respiratory diseases to squill's anticholinergic and anti-inflammatory actions [42]. Squill compounds possess immunomodulatory effects much more potent than conventional immunosuppressive drugs and steroids, and squill has antioxidant and bronchodilatory effects.

Abolghasemi et al. mentioned the promising effects of *Zataria multiflora* Boiss on insulin resistance [37]. They showed that simple oxymel and thyme oxymel might reduce insulin resistance and its levels in high doses.

### 4.5 Effects of adjuvant therapies

Adjuvant therapies used alongside oxymel included cupping in one article and sauna (using a defined dry heat chamber) in three articles. In such studies, caution should be exercised when evaluating the observed therapeutic effects. Firoozabadi et al. attributed the analgesic effects of the simultaneous administration of oxymel and cupping to several possible mechanisms, including the regulation of neurotransmitters involved in headaches, and the adjustment of hematocrit and immunomodulation [56].

In three animal studies, the concomitant use of oxymel with sauna was assessed. This protocol is called SINA therapy, a novel therapeutic method derived from the principles of PM. Since SINA therapy was the central investigated intervention in these researches, animals were grouped into oxymel, sauna, and SINA therapy (both oxymel and sauna). The findings of these studies show different effects for each of these treatment modalities and a cumulative adjuvant effect of sauna and oxymel in SINA therapy [39,43,49].

### 4.6 PM's point of view

While observed therapeutic effects are generally interpreted based on oxymel ingredients, some articles justify the mechanisms of action via PM's principles. According to PM's perspective, the temperaments of vinegar and honey/sugar generally reach equilibrium during cooking and concentration. This process leads to new features that do not exist in the two primary components alone, resulting in new medicinal and nutritional values with unique properties.

Some of the most critical actions and properties mentioned for oxymel in PM sources include the separation and excretion of sticky viscous substances (scraper: *Moqatte’*), the dilution of blood and humors (attenuant: *Molattef*), and the opening of obstructions (deobstruent: *Mofatteh*) [17,19,27]. Based on these mechanisms, PM textbooks have recommended oxymel to manage problems such as blood thickness, thick sticky sputum and severe coughs associated with it, and obstructions of vessels or ducts (such as bile ducts) [18,19].

Moreover, oxymel can be used as an emetic, diuretic, expectorant, and laxative agent when there is a need to eliminate abnormal substances from the body. An example of such use is in critically-ill patients who are on the verge of the critical stage of the disease, in which the physician predicts the future occurrence of a sudden excretion (as in the critical days of the disease) based on prodromal signs and symptoms. Thus, oxymel is prescribed to facilitate the process [15,18,32,33].

In one of the primary PM sources on single drug monographs, *Makhzan al-Advieh*, written by *Aghili Shirazi*, oxymel is mentioned as a corrigent (*Mosleh*) for many medicinal substances [17]. According to statistics from the UNaProd database [57], it is the second most frequent medicine used to modify drug actions or counteract their adverse effects.

Latifi et al. explained pathophysiological symptoms of prostate pain syndrome based on PM principles. They justified the effect of oxymel in relieving symptoms via improving digestion and discussed its anti-flatulence and diuretic properties [52].

Regarding PM literature, Nejatbakhsh et al. described asthma as a phlegmatic disorder and squill as one of the best modifiers of phlegmatic dystemperament. PM sources refer to simple honey oxymel and squill oxymel as a treatment for asthma, although the latter is considered a richer and more potent formulation. According to PM, the positive effect of simple honey oxymel is due to its modulatory effects on the temperament of the respiratory system [42].

Denoting the PM viewpoint, Sarbaz Hoseini et al. justified the observed increase in insulin bioavailability and decrease in insulin resistance to the actions of oxymel on blood thinning, tissue cleansing, and increasing tissue porosity and permeability. On the other hand, external heat causes peripheral vasodilation. Administered simultaneously with oxymel (SINA therapy protocol), it can improve general blood flow to various body organs, including the pancreas, and induce regeneration of the islets of Langerhans [39,49].

The combination of oxymel with thermal therapy, derived from the principles of PM, has beneficial effects on the heart and blood circulation. PM literature emphasizes that this intervention helps the body repair itself using its inherent power [43].

### 4.7 Oxymel's advantages

Oxymel offers numerous advantages compared to other functional foods. The combination of vinegar and honey in oxymel can help improve digestion. For instance, vinegar, has been shown to improve digestion and increase satiety. This can be beneficial for individuals with diabetes who often struggle with digestion issues and weight management. Improved digestion can also positively impact blood sugar control. In addition, vinegar contains acetic acid, which stimulates the production of digestive enzymes, aiding in the breakdown of food [[58], [59], [60], [61]]. Honey, on the other hand, has prebiotic properties, promoting the growth of beneficial gut bacteria and supporting a healthy digestive system [[62], [63], [64], [65]]. Additionally, this formulation is not only natural but also deeply rooted in tradition. Oxymel has been used for centuries as a natural remedy for various health issues. It is made from simple, readily available ingredients and does not contain any artificial additives or preservatives. This traditional preparation method ensures that the product is natural and safe for consumption. Another crucial aspect is the versatility of Oxymel. By utilizing a variety of herbs, it offers a broad spectrum of health benefits. Each herb provides its unique properties, making it a versatile functional food that can address various health concerns. For example, oxymel made with ginger is known to aid digestion [66]. The ease of consumption should be pointed as another important point. Oxymel is easy to consume, making it a convenient functional food. It can be taken directly by the spoonful or added to beverages like water or tea. The combination of vinegar and honey also provides a pleasant taste, making it more palatable compared to other functional foods. Moreover, oxymel can be prepared at home using simple ingredients, making it a cost-effective functional food option. Buying commercial functional foods or supplements can be expensive, whereas making oxymel at home allows for customization and reduces the overall cost.

The use of oxymel in the management of chronic diseases offers several potential benefits compared to conventional medications. For instance, oxymel has antioxidant and immune-boosting properties. In addition, conventional medications often carry a range of potential side effects, while oxymel is generally well-tolerated and associated with minimal side effects, making it a potentially safer option for long-term use in managing chronic diseases. It should be noted that oxymel not only addresses the symptoms of chronic diseases but also supports overall health and wellness, taking a more holistic approach to managing chronic conditions. Patient compliance is another important consideration in the management of chronic diseases, and it is possible that oxymel may offer advantages in this area compared to conventional medications. One potential advantage is that oxymel is made from natural ingredients, which may be more appealing to patients who are concerned about the use of synthetic chemicals and additives in their treatment. Additionally, the customizable nature of oxymel may allow patients to have more control over their treatment and tailor it to their individual needs, potentially increasing their motivation to comply with treatment recommendations.

### 4.8 Future directions

In the context of differing effects of honey and sugar, as interpreted in PM due to variances in their temperaments, studies can be structured to compare the effects of sugar oxymels and honey oxymels.

Oxymel has a long history of use in managing signs, symptoms, and specific conditions in critically ill patients. It possesses important properties, such as opening vascular and ductal blockages, diluting blood, and facilitating expectoration. Therefore, it is feasible to design studies to investigate the cautious use of this food-drug in appropriate doses in the diets of critically ill and intubated ICU patients, provided contraindications and drug interactions are considered.

Simple oxymels may be beneficial for all temperament groups due to their mild temperament [67]. Therefore, due to this and its disease prevention properties, such as preventing blockage or inflammation, the drink has been recommended for long-term consumption in both healthy individuals and patients [19]. Recent studies also suggest that, as a beneficial drink for healthy individuals, oxymel can replace harmful industrial beverages [45,46].

## 5 Conclusion

Oxymel appears to be not only a functional drink to maintain health and prevent disease in healthy individuals but also an effective and safe drug in the treatment or management of several diseases, including T2DM, obesity, and asthma. This drink can serve as a base for some formulas or in the production process of certain drugs. Although no serious side effects have been reported in the reviewed studies, it is important to note that interventions and follow-ups were not long-lasting. Therefore, further research with larger sample sizes is needed to investigate the possible side effects of long-term oxymel use, as well as potential interactions with other drugs.

## Funding

This study was financially supported by a grant from 10.13039/501100004320Shiraz University of Medical Sciences (grant No. 26739).

## Data availability statement

Data used to support the findings of this study are included within the article.

## CRediT authorship contribution statement

**Narges Sharifi Darani:** Writing – original draft, Methodology, Investigation, Conceptualization. **Mahdi Alizadeh Vaghasloo:** Writing – review & editing, Methodology. **Asma Kazemi:** Writing – review & editing, Methodology. **Hakima Amri:** Writing – review & editing, Methodology. **Thomas Rampp:** Writing – review & editing, Methodology. **Mohammad Hashem Hashempur:** Visualization, Supervision, Methodology, Investigation, Conceptualization, Funding acquisition, Writing - original draft.

## Declaration of competing interest

The authors declare that they have no known competing financial interests or personal relationships that could have appeared to influence the work reported in this paper.
